# COVID-19 Vaccination-Induced Rash: Does the Choice of Vaccine Matter?

**DOI:** 10.7759/cureus.15490

**Published:** 2021-06-07

**Authors:** Mansoor Zafar, Baby Ewnetu, Saif Ahmed, Uzair Iqbal, Mark Whitehead

**Affiliations:** 1 Gastroenterology and Hepatology, Conquest Hospital, East Sussex Healthcare NHS Trust, St. Leonards-on-Sea, GBR; 2 Acute Care Common Stem: Anaesthesia-General Internal Medicine-Emergency Medicine, Conquest Hospital, East Sussex Healthcare NHS Trust, St. Leonards-on-Sea, GBR; 3 General Internal Medicine, Conquest Hospital, East Sussex Healthcare NHS Trust, St. Leonards-on-Sea, GBR; 4 Acute Medicine, General Internal Medicine, Conquest Hospital, East Sussex Healthcare NHS Trust, St. Leonards-on-Sea, GBR; 5 Gastroenterology, Conquest Hospital, East Sussex Healthcare NHS Trust, St. Leonards-on-Sea, GBR

**Keywords:** covid 19, covid-19 vaccine, drug rash, absolute eosinophil count, healthcare policies

## Abstract

With the introduction of large-scale vaccination programmes against the coronavirus disease 2019 (COVID-19), the world has now begun to visualise a possible end to the ongoing pandemic. As with any vaccination programme, reports of side effects have begun to emerge in the wake of vaccinations. Initial reports were about mild side effects, such as local inflammation, pain, and fever. However, as a significant number of the population began to receive various COVID-19 vaccines, reports of various other moderate to severe side effects have now started to emerge. Although these side effects seem to be rare, the symptoms can be severe, and information and guidelines on how to manage them are scarce. In this case series, we discuss the incidence of widespread rashes that develop in some individuals after receiving COVID-19 vaccines by both AstraZeneca (AstraZeneca plc, Cambridge, UK) and Pfizer-BioNTech (Pfizer Inc., Brooklyn, NY; BioNTech SE, Mainz, Germany). The systemic skin reaction varied from maculopapular rashes to papules and patches that were widespread and not simply localised to the vaccine injection site. Further clinical information, awareness, and guidelines for practicing clinicians need to be exigently provided as vaccination programmes approach completion and the incidences of moderate to severe side effects of COVID-19 vaccination are becoming more apparent and pervasive.

## Introduction

The introduction of coronavirus disease 2019 (COVID-19) vaccines has provided a huge sense of relief to the global population rattled by this pandemic that has already resulted in millions of deaths worldwide. However, since the introduction of these vaccines, there has been a number of reports related to their harmful side effects. Very little was known about COVID-19 when it first emerged in China and, similarly, reliable and authoritative information on the consequences of COVID-19 vaccinations remains scarce, and no guidelines have yet been provided with regard to managing them. One registry has reported on cutaneous manifestations in patients after being vaccinated with Pfizer-BioNTech (Pfizer Inc., Brooklyn, NY; BioNTech SE, Mainz, Germany) or Moderna (Moderna, Inc, Cambridge, MA) vaccines in the United States (US) [1]; although their focus was mainly on cutaneous manifestations, we feel that they did not provide a complete picture of the scenario.

In the United Kingdom (UK), the vaccines administered have included those manufactured by Pfizer-BioNTech and AstraZeneca (AstraZeneca plc, Cambridge, UK). The vaccination of people in large numbers has led to speculations and reports about vaccine-related side effects such as cutaneous manifestations post-vaccination. Initially, the National Institute for Health and Care Excellence (NICE) guidelines in the UK and those issued by the Centers for Disease Control and Prevention (CDC) in the US did briefly touch on potential reactions at the site of vaccination. However, in acute clinical practice, the clinicians have faced a number of varied situations regarding cutaneous manifestations. These include disseminated skin reactions, with varying times of onset, as well as varying D-dimer levels and blood eosinophil counts. Fortunately, with the support of our consultants, we have been able to manage the patients coming to the acute clinic after being referred to from the emergency department of our hospital. Although we have reported via the Medicines and Healthcare products Regulatory Agency's (MHRA) national Yellow Card Scheme regarding certain adverse events, our paramount objective has been to ensure safe practices combined with overall patient well-being. In this report, we attempt to outline how the patients are managed and emphasise the urgent need to update the World Health Organisation (WHO) guidelines about vaccine side effects, as well as those by NICE in the UK and CDC in the US. This will not only help the practicing clinicians to be more confident but will also educate the masses and help relieve their anxieties related to vaccine-related side effects, which have often made them panic and rush to the hospital emergency departments. To the best of our knowledge, this report is the first of its kind in medical literature in terms of findings related to cutaneous and blood result manifestations, and we have endeavoured to connect the dots pertaining to vaccine side effects. Another major aim of this report was to highlight the importance of managing patients with widespread disseminated skin reactions with varying times of onset and varying symptoms and blood test results.

## Case presentation

Case 1

An 84-year-old male, who was independently mobile but with a history of benign prostatic hypertrophy, presented to the Emergency Department accompanied by his wife with widespread disseminated mildly itchy rash, which had started 11 days after his second dose of COVID-19 vaccine (BNT162b2, Pfizer-BioNTech). His initial blood D-dimers were 2,250 ng/ml (normal range: 0-225 ng/ml), fibrinogen level was 3.9 g/L (normal range: 1.8-3.6 g/L), platelet count was 146 x 10^9^/L (normal range: 150-400 x 10^9^/L), eosinophils were 0.49 x 10^9^/L (normal range: 0-0.4 x 10^9^/L), prothrombin time (PT) was 10.9 seconds (normal range: 10-11.7 seconds), and his international normalised ratio (INR) was 1. Immunoglobulins, vasculitis, hepatitis, and HIV screen were negative. Complement C3 was 1.46 g/L (normal range: 0.90-1.80 g/L), and C4 was 0.25 g/L (normal range: 0.10-0.40 g/L). Repeat tests the next day revealed D-dimer of 1,112 ng/ml, fibrinogen of 3.4 g/L, platelet count of 144 x 10^9^/L, eosinophils of 0.68 x 10^9^/L, PT of 10.6 seconds, and INR of 1. He was advised to take oral antihistamines and topical steroids with follow-up in the ambulatory clinic. His subsequent review in the ambulatory clinic showed near resolution of his rash, along with near normalisation of his blood test results. He was discharged back to the care of his general practitioner, with outpatient follow-up with dermatology.

**Figure 1:**
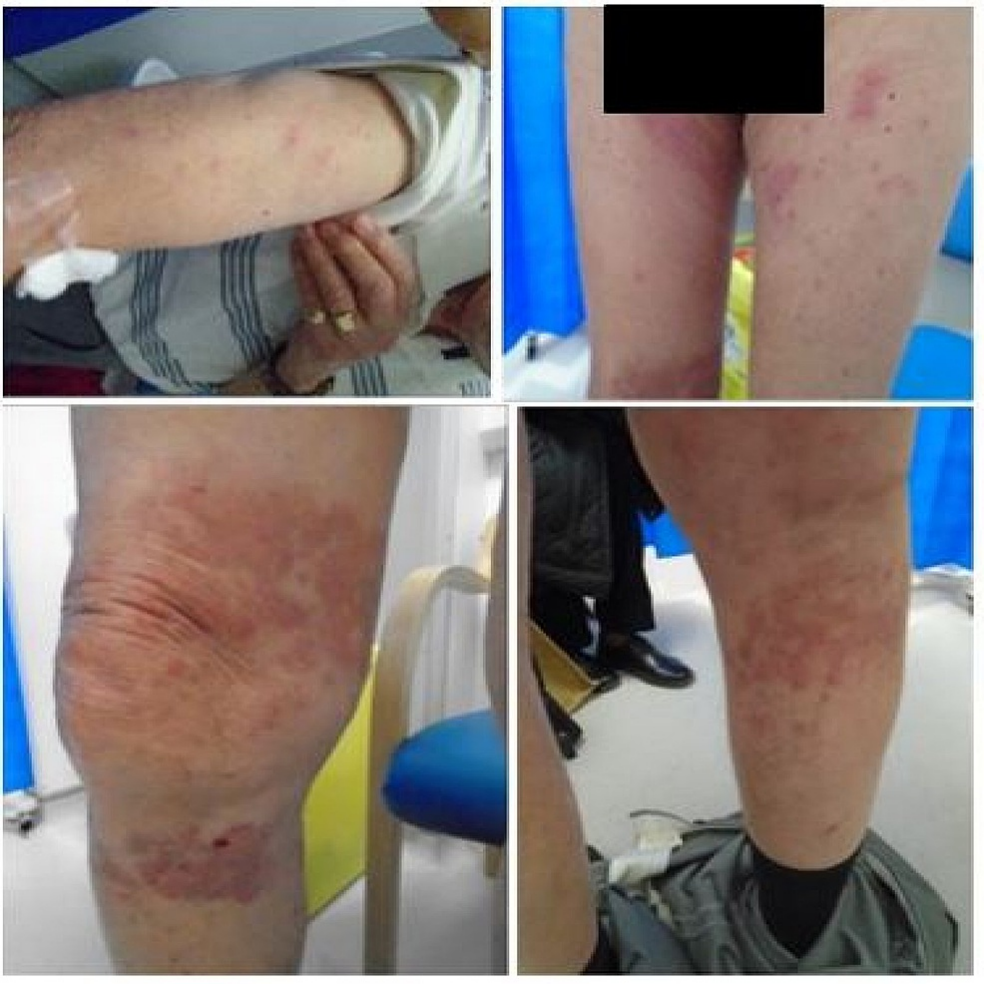
Appearance of rash post-COVID-19 vaccination (BNT162b2, Pfizer-BioNTech)* *30 micrograms/0.3 ml dose concentrate for suspension for injection multidose vials (Pfizer Inc.) COVID-19: coronavirus disease 2019

Case 2

A 55-year-old female with a history of stable psoriasis and scleroderma was referred by the general practitioner for new-onset non-itchy rashes that had appeared two days after her first dose of AstraZeneca/Oxford COVID-19 [ChAdOx1 S (recombinant)] vaccine; it had progressively worsened over the past one month on her hands and back. Blood tests showed a platelet count of 143 x 10^9^/L, eosinophils of 0.07 x 10^9^/L, PT of 11.4 seconds, and INR of 1.1; immunoglobulins, hepatitis, and HIV screening were negative. Complement C3 was 1.10 g/L, C4 was 0.13 g/L, fibrinogen level was 2.4, and D-dimer was 274 ng/ml. Her repeat blood test showed a platelet count of 121 x 10^9^/L, eosinophils of 0.01 x 10^9^/L, D-dimer of 235 ng/ml, PT of 11.1 seconds, and INR of 1.1. She declined admission and agreed to attend outpatient review in the dermatology clinic; she was subsequently found to be recovering from the rash and her blood test results subsequently normalised while on oral antihistamines and topical steroid creams.

**Figure 2:**
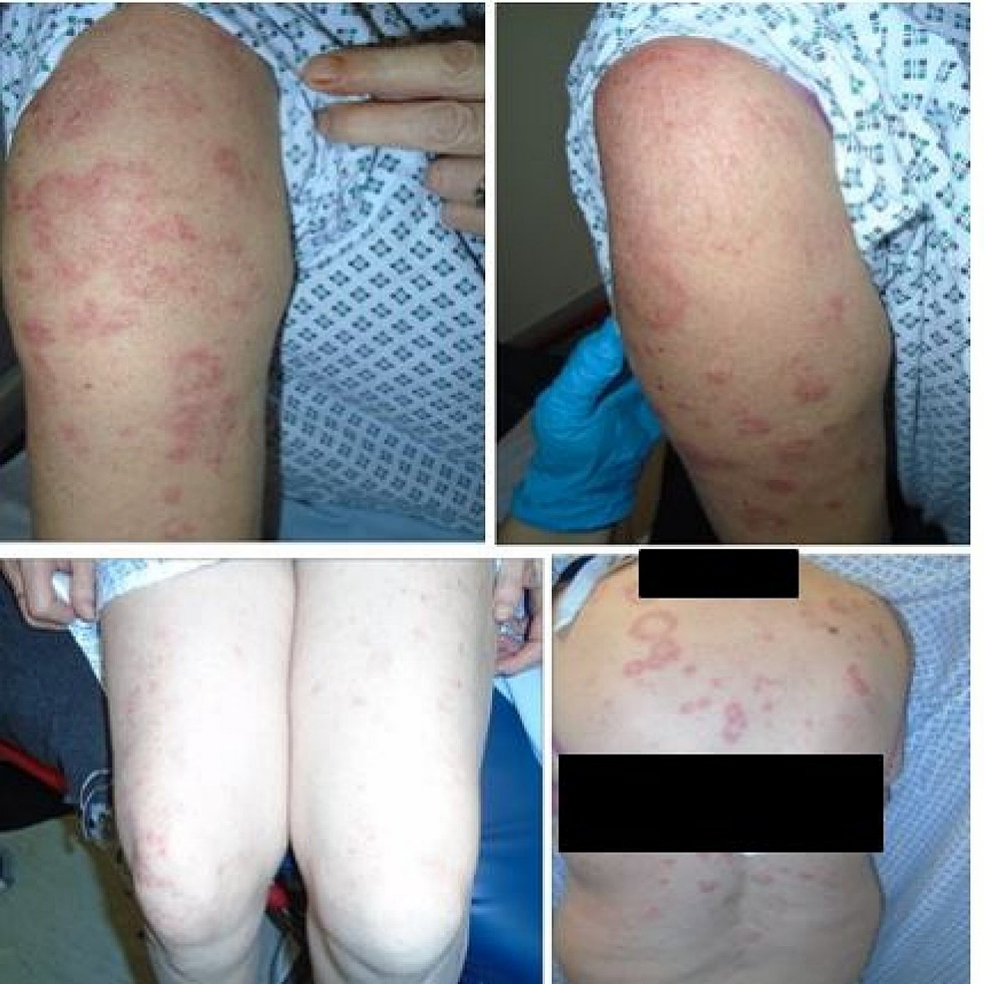
Appearance of rash post AstraZeneca/Oxford COVID-19 [ChAdOx1 S (recombinant)] vaccination* *5 x 10,000,000,000 viral particles/0.5 ml dose solution for injection multidose vials (AstraZeneca plc) COVID-19: coronavirus disease 2019

## Discussion

As the world struggles with the effects of the COVID-19 pandemic, the news of the emergence of vaccines against the condition provided a real sign of hope [2]. Since the start of the massive vaccination programme in early December 2020, 175.3 million vaccine doses have been administered as of February 15, 2021. WHO issued an Emergency Use Listing (EULs) for the Pfizer-BioNTech COVID-19 vaccine (BNT162b2) on December 31, 2020, and on February 15, 2021, WHO issued EULs for two versions of the AstraZeneca/Oxford COVID-19 vaccine [2]. In an effort to inform the people about the side effects of the vaccines, the National Health Service (NHS) and NICE in the UK initially notified the public about the initial side effects reported, including the incidence of a sore arm where the needle went in when the individual was feeling tired, headaches, feeling achy, and a general feeling of being sick [3,4]. However, soon after that, other side effects began to be widely reported, including the development of venous thromboembolism [5] or thrombocytopenia [6]. The development of skin rashes was another newer side effect reported [7]. However, it has been suggested that people who develop rashes, especially after their first dose of vaccine, need not worry much and should still consider getting their second dose of vaccine, although this view has been contested. The exact cause of DVT or skin rashes is still a matter of debate [5,7].

McMahon et al. have conducted a study regarding post-vaccination rash in 414 patients who reported symptoms of rash. This study compared the rashes developed among those who took the Pfizer-BioNTech (BNT162b2) vaccines with those who received the Moderna (mRNA-1273) COVID-19 vaccine. They reported the median age of the onset of rash to be seven days post-vaccination for Pfizer-BioNTech and eight days for Moderna vaccines. Unfortunately, they only recorded cutaneous manifestations, and hence the valuable finding of eosinophilia as reported in one of our patients had not been described previously [1]. The CDC in the US has recommended that If one experiences “COVID arm” after getting their first shot, they should still get the second shot after the recommended interval if the vaccine they received warrants a second shot, and should notify the vaccination provider about the rash or “COVID arm” after the first shot. CDC recommends that the vaccination provider may advise such people to get their second shot in the opposite arm [8]. However, the guidelines are not clear about cases of widespread rash. This issue has become more relevant now as the Department of Health and Social Care (DHSC) in the UK has announced the rollout of a COVID-19 booster vaccine starting at the beginning of autumn in order to protect the most vulnerable sections of the population ahead of the winter [9]. 

With the administration of COVID-19 vaccines, patients develop adverse side effects in varying intensities and frequencies, which are usually either allergic or a delayed hypersensitivity reaction. With the expansion of the vaccination drive, more and more adverse effects are surfacing, and these include effects that have not been commonly reported so far. Cutaneous adverse reactions are one of the possible side effects of the vaccine. As practicing physicians, we have to promptly identify them in order to not over-treat the patients and, over a period of time, should come up with recommendations for targeted vaccination strategies based on patient demographics and morbidity.

We believe this case series provides a good base to launch a more detailed study focusing on short-term and long-term adverse effects of COVID-19 vaccines. Ultimately, it is all about the risk-benefit ratio. As physicians, we can make an educated decision only if we were well-informed about all the adverse effects that a patient can develop due to vaccinations; hence, we feel our efforts will likely encourage others toward documenting further cases related to the issue, which would hopefully provide deeper insights into the causes of the various side effects.

In this series, we reported two cases that presented with adverse cutaneous reactions to the COVID-19 vaccine. We attempted to outline delayed onset of rash in association with eosinophilia 11 days post-Pfizer vaccination versus worsening rash with normal eosinophil count one-month post-AstraZeneca vaccine in a patient with a history of comorbid autoimmune disorder. We hope that our findings may provide a missing piece to the puzzle of allergic versus delayed-onset reaction with or without eosinophilia in individuals who have received the COVID-19 vaccine.

## Conclusions

Symptomatic management with antihistamine oral medications and topical application of steroid creams for a short duration have shown good results in the treatment of side effects of the COVID-19 vaccine. Since the incidence of widespread rashes seems to be occurring more frequently, there is a need to update NICE and CDC guidelines regarding the management of disseminated widespread rash post-COVID-19 vaccination, and there is also a need to raise more awareness about its management. In particular, there is a critical need to update the guidelines regarding side effects following the first dose of the COVID-19 vaccination, especially in light of the recent plans to roll out booster vaccination during the upcoming autumn season.
